# Synthesis, Purification, and Physical Properties of Seven Twelve-Carbon Hydrocarbons

**DOI:** 10.6028/jres.067A.049

**Published:** 1963-10-01

**Authors:** Thomas W. Mears, Connie L. Stanley, Edward L. Compere, Frank L. Howard

## Abstract

As part of a program to determine accurately the heats of combustion of specialized fuels in the kerosene range, seven hydrocarbons, biphenyl, bicyclohexyl, cyclohexylbenzene, *n*-hexylbenzene, *n*-hexylcyclohexane, 1-cyclopentylheptane, and *n*-dodecane were synthesized or purified from commercial material. Physical constants were determined on the purified samples. These materials may have use as secondary standards for heat of combustion measurements.

## 1. Introduction

These syntheses and purifications are a part of the project concerned primarily with heats of combustion and other properties of kerosene-like fuels. For certain specific applications it. is necessary not only to know the heat of combustion of bulk lots of material but to have very exact knowledge of this property for individual portions of the bulk immediately before use. As an aid to the precise determination of these heats of combustion, it was decided to synthesize a series of typical hydrocarbons.

Since the fuels of interest are either kerosenes per se or hydrocarbon mixtures boiling in the kerosene range, it was decided that the materials synthesized should have molecular weight encompassed by the kerosene range and the hydrocarbon types represented should be those present in some or all commercial kerosenes. Since most kerosenes distill between 175 and 260 °C, it was decided that the most characteristic hydrocarbons would be those containing 12 carbon atoms. Therefore, 7 hydrocarbons of different structures, but all containing 12 carbon atoms, were selected for synthesis and purification. Three classes of compounds containing 12 carbon atoms typical of hydrocarbons found in kerosenes were not included, namely, dimethyldecalins, dimethyltetralins, and dimethylnaphthalenes, as they either boil above the kerosene range or are present only in small amounts.

## 2. Synthesis and Purification of the Hydrocarbons

The hydrocarbons were, in general, synthesized by modifications of techniques used earlier [2, 3]^2^ these methods involved the synthesis of an appropriate carbinol, the dehydration of this carbinol to an olefin or a mixture of olefins, and the catalytic hydrogenation of the olefins to paraffins.

The liquid hydrocarbons were purified by fractional distillation. Two types of distillation apparatus were used. One was a 6 ft by 1 in. column described by Whitmore and Lux [4] packed with 316-in. glass helices. The head was a modification of the Whitmore-Lux type designed by Howard [5]. These columns had an efficiency of 25 to 30 theoretical plates at atmospheric pressure and were used largely in the separation and purification of intermediates. The other column was a 3 ft by 25 mm Podbielniak “Heli-grid” column [6] having an efficiency of about 85 theoretical plates at atmospheric pressure, and was used for the final distillations. Both columns were equipped to operate at reduced pressures.

### 2.1. Purification of Biphenyl

Biphenyl, obtained by purchase,^3^ had a purity of 99.86 to 99.88 mole percent [1, 7] and was further purified by the single crystal technique of Horton and Glasgow [8, 9].

The biphenyl was distilled into the crystallization tube and sealed under its own vapor pressure. The tube was then lowered slowly into a bath containing two immiscible liquids at different temperatures. The upper liquid was a silicone oil heated to 95 °C, the lower liquid was ethylene glycol held at 45 °C. The interface between these two liquids provided a sharp, plane boundary between the two temperature zones. As the sample passed from the warmer to the cooler zone, the biphenyl slowly crystallized with the impurities concentrating in the liquid phase. The rate of crystallization was ½ to 1½ in. per day with a total time of 10 to 25 days for a single crystallization. The crystallization tubes were so designed that the small amount of residual liquor remaining near the end of a crystallization could be removed without exposing the main sample to air. The crystal could then be melted and the process repeated. Each sample was crystallized three times.

The purity of these samples was determined by a modification of the freezing point method of Glasgow, Streiff, and Rossini [10]. The freezing points, 68.931 °C to 68.951 °C, correspond to purities of 99.92 to 99.96 mole percent [7].

### 2.2. Purification of Cyclohexylbenzene

**Figure 1:**
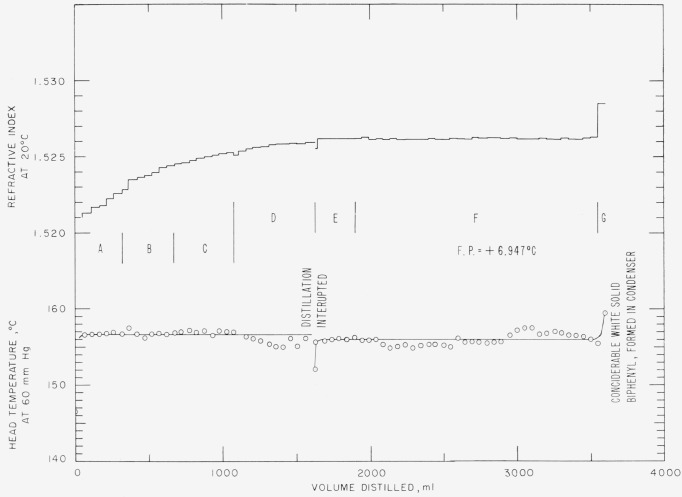
Distillation of cyclohexylbenzene, charged—3750 ml.

Cyclohexylbenzene was obtained by purchase^4^ and purified by fractional distillation at a pressure of 60 mm Hg using the “Heli-grid” column. Fractions of approximately 50 ml each were collected and the refractive indices determined. The individual fractions were grouped into cuts largely on the basis of uniformity of refractive indices. The data shown in figure 1 are typical of the three distillations of cyclohexylbenzene. The physical properties measured on cuts E and F are given in table 1. Cuts C and D were subsequently hydrogenated to bicyclohexyl (see section 2.3.).

Other methods of purification were attempted. One was the slow, fractional melting of solidified cyclohexylbenzene and separation in a specially designed basket centrifuge [11]. The crystals were placed in the basket of the centrifuge which was held at a temperature near the melting point of the crystals. The basket was rotated rapidly and, as the crystals slowly melted, the liquid was thrown from the basket and collected into fractions. The refractive index and freezing point of each fraction were determined. This method was not satisfactory, probably because of the relatively low heat of fusion of cyclohexylbenzene, or possibly the formation of solid solutions. A second method used was chromatographic adsorption on alumina. Commercial cyclohexylbenzene of 92 percent purity, when passed once through an alumina column, showed a 2.5 to 3.0 percent increase in purity of some of the eluent fractions. Material which had been previously purified by fractionation and crystallization to a purity of 98.65 percent was improved to 99.12 percent in one pass and to 99.24 percent in a second pass. These improvements, however, were accompanied by a high loss of material. Attempts to apply silica gel chromatography to the purification of this material proved completely unproductive.

### 2.3. Synthesis and Purification of Bicyclohexyl

Bicyclohexyl was prepared by catalytic hydrogenation of selected fractions from the distillations of cyclohexylbenzene. The fractions used for hydrogenation were those immediately preceding the material collected as purified cyclohexylbenzene. The hydrogenation was carried out using nickel-on-kieselguhr catalyst at 180 °C±10 °C and an initial pressure of about 1850 lb/in.^2^

The hydrogenated hydrocarbon was filtered to remove the catalyst and percolated through silica gel prior to distillation. The material boiling at 148.5 to 150 °C at 58 mm Hg with refractive indices at 20 °C of 1.4796 to 1.4797 (uncorr.) was collected as bicyclohexyl. The product was again percolated through silica gel and certain physical properties were measured.

### 2.4. Synthesis and Purification of 1-Cyclopentyl- heptane

The 1-cyclopentylheptane was prepared in two ways. In the first, 1-heptyi-1-cyclopentanol was synthesized. Magnesium turnings, (53 g, 2.2 moles) were covered with ether and 296 g (2.2 moles) of *n*-heptyl chloride in 500 ml of ether was slowly added. This was followed by the slow addition of 168 g (2 moles) of cyclopentanone in 200 ml of ether. After the mixture had been stirred for 2 to 3 hr, it was hydrolyzed and the ether removed in the usual manner. The products from several runs were combined and distilled at reduced pressure. However, only a small amount of 1-heptyl-1-cyclopentanol (bp 91 to 92 °C at 3 mm Hg, $n_{D}^{20}$ 1.4604 [12]) was isolated. This was due to the extensive dehydration that took place during distillation. The partially dehydrated material was distilled rapidly at atmospheric pressure to give a fraction boiling between 218 and 220 °C. This material contained a considerable amount of the carbinol along with the olefin resulting from the dehydration. An approximate estimate of the combined yield of these compounds is 20 percent. The low yield is probably due to the ease with which cyclopentanone tautomerizes to the enol form. The carbinol-olefin product was passed over alumina at 300 °C to complete the dehydration.

To conduct the synthesis by another path, 1 -cyclopentyl-1-heptanol was prepared from cyclopentyl- magnesium bromide and *n*-heptaldehyde in a similar manner. The Grignard reagent was prepared by reacting 328 g (2.2 moles) of cyclopentyl bromide with 53.5 g (2.2 moles) of magnesium turnings in 600 ml of dry ether. To this was added 228 g (2 moles) of redistilled *n*-heptaldehyde (bp 152 to 157 °C) in 270 ml of ether. The usual procedure of hydrolysis, washing, drying, and removal of the ether was followed. The residues from 14 runs (28 moles of *n*-heptaldehyde) were combined and distilled in two charges in the glass-helix packed column. The major fractions were 1-heptanol (bp 102.4 to 113.4 °C at 50 mm Hg, $n_{D}^{20}$ 1.4260 to 1.4242) 1300 g, and 1-cyclopentyl-1-heptanol (bp 168 to 170 °C at 60 mm Hg, $n_{D}^{20}$ 1.4606 to 1.4611) 1944 g. The reduction of *n*-heptaldehyde by the Grignard reagent to 1-heptanol accounts for 41 percent of the aldehyde used. The yield of 1-cyclopentyl-1-heptanol was 38 percent.

The 1-cyclopentyl-1-heptanol was dehydrated by passage over alumina at 265 to 300 °C. Approximately 90 percent of the theoretical quantity of water was recovered. The crude olefin was washed, dried, and distilled in the glass-helix packed column. The material distilling between 132 and 134 °C at 54 mm Hg ( $n_{D}^{20}$ 1.4565 to 1.4571), was collected as the mixed 1-cyclopentylheptenes. The fractions of higher boiling point and refractive index containing undehydrated carbinol were recycled through the dehydration apparatus. The overall yield was 84 percent.

The incompletely dehydrated material from the reaction of *n*-heptylmagnesium chloride and cyclopentanone was also dehydrated over alumina at 265 to 300 °C. The olefin mixture *was* hydrogenated with nickel-on-kieselguhr catalyst at 130 °C and hydrogen pressure not exceeding 1200 lb/in.^2^ The hydrogenation proceeded smoothly up to a point of partial hydrogenation after which no more hydrogen was absorbed. In order to complete the hydrogenation it was necessary to raise the temperature to 160 °C and the pressure to 2000 lb/in.^2^ The hydrogenated product was washed, dried, and percolated through a silica gel column.

The 1-cyclopentylheptane was purified by fractionation on a 3-foot “Heli-grid” [5] column under approximately 50 mm Hg pressure. The purification was made difficult by a small amount of an unidentified material with a boiling point near that of the product. This made it necessary to redistill the heart cuts of several distillations before obtaining a sample of satisfactory purity. The final distillation gave material boiling 142.1 to 142.9 °C at 50 mm Hg.

### 2.5. Synthesis and Purification of *n*-Hexylbenzene

Normal-hexylbenzene was prepared by a method similar to that used in the synthesis of 1-cyclopentylheptane. Sufficient *n*-hexylbenzene was prepared so that a part of it could be further hydrogenated to *n*-hexylcyclohexane. In a typical run a Grignard reagent was prepared by the reaction of 415 g (2.75 moles) *n*-amyl bromide (bp 127 to 129 °C) in 800 ml of ether with 66.9 g (2.75 moles) of magnesium turnings. To this was slowly added 265 g (2.5 moles) of benzaldehyde in 250 ml of ether. During the course of the reaction, it was necessary to add ether to keep the reaction fluid. After the addition was complete the reaction mixture was refluxed for 2 hr, and hydrolyzed with saturated ammonium chloride solution. The ether layer was siphoned off and the aqueous layer extracted four times with ether. The ether extracts were combined, dried, and the ether removed by distillation.

The crude products from four Grignard reactions (9 moles of benzaldehyde) were combined and distilled under reduced pressure in the glass-helix packed column. The fractions boiling at 172 °C at 50 mm Hg, $n_{D}^{20}$ 1.5064 to 1.5048 [13], were collected as 1-phenyl-1-hexanol. The yield was 1264 g (7.1 moles) which represents 79 percent yield on the basis of benzaldehyde used; a total of 4.5 kg was prepared.

The 1-phenyl-1-hexanol was successfully dehydrated over alumina at temperatures ranging from 270 to 315 °C. Since the dehydration resulted in some movement of the double bond to produce a series of close-boiling olefins no attempt was made to isolate individual compounds. The crude olefin mixture was separated from residual carbinol compounds by distillation under reduced pressure (10 to 20 mm Hg) using a short Vigreaux column. The entire lot of mixed phenylhexenes was hydrogenated over nickel-on-kieselguhr catalyst at 120 °C and pressures up to 1600 lb/in.^2^ and the amount of hydrogen fed into the reactor was measured. Under these conditions, the absorption of hydrogen ceased upon the saturation of the olefinic double bond without attacking the benzenoid nucleus. Distillation of the *n*-hexylbenzene in the “Heli-grid” column under reduced pressure gave several fractions boiling from 132 to 133 °C at 47 mm Hg, $n_{D}^{20}$ 1.4866 to 1.4867. A portion of this material was reserved as *n*-hexylbenzene and the balance along with selected foreruns was further hydrogenated to *n*-hexylcyclohexane.

### 2.6. Synthesis and Purification of *n*-Hexylcyclohexane

Normal hexylcyclohexane was prepared by the hydrogenation of *n*-hexylbenzene (5) using nickel-on- kieselguhr catalyst. The temperature of the hydrogenator was raised to 180 to 190 °C and the peak pressures were about 2100 lb/in.^2^ The hydrogenated product was percolated through silica gel and distilled.

Distillation of the product in the 3 ft “Heli-grid” column under reduced pressure gave two principal cuts: cut C (bp 144 to 147 °C at 65 mm Hg, $n_{D}^{20}$ 1.4471 to 1.4469), 867 ml; and cut D (bp 147 °C at 65 mm Hg, $n_{D}^{20}$ 1.4468 to 1.4465), 707 ml. Both cuts were percolated through silica gel and their freezing points determined. Cut C gave a freezing point of −47.51 °C and was reserved as “best” material. Cut D was less pure with a freezing point of −51.77 °C.

### 2.7. Synthesis and Purification of *n*-Dodecane

*n*-Dodecane was prepared by the hydrogenation of the olefins obtained from the dehydration of lauryl alcohol. Lauryl alcohol (1-dodecanol) was dehydrated by passage over alumina at 350 to 375 °C with a yield for a single pass of about 80 percent. The organic liquid was washed, dried, and fractionated through a short, helix-packed, distilling column. The material boiling from 210 to 215 °C was collected as mixed dodecenes; and the residue was recycled through the dehydration apparatus. The overall yield of olefin based on dodecanol consumed was about 88 percent.

The mixed dodecenes were hydrogenated with nickel-on-kieselguhr catalyst at 140 °C and initial hydrogen pressures of 1800 lb/in.^2^ The *n*-dodecane was freed from nickel and percolated through silica gel. The final product was fractionated in the glass- helix-packed column. The fractions boiling at 126 °C at 50 mm, $n_{D}^{20}$ 1.4218 to 1.4219 were collected as *n*-dodecane.

## 3. Determination of Physical Properties

Physical properties and purities were determined on the best samples of each hydrocarbon, and the values obtained are given in table 2. Boiling points, densities, and refractive indices were determined by the procedures described by Mears et al. [3]. The constant temperature apparatus was maintained within ±0.02 °C during the measurement of density and refractive indices.

The freezing points and purities were determined where possible by the method of Glasgow, Streiff, and Rossini [10]. The freezing point curves obtained using the monocyclic hydrocarbons with long side chains gave purity values which seemed incompatible with estimates of purity based on other properties. For this reason the estimates of purities of 1-cyclopentylheptane, *n*-hexylcyclohexane, and *n*-hexylbenzene were determined from vapor-phase chromatograms. In each case only one peak was noted.

It has been observed earlier in this laboratory that the freezing points of long-chain normal hydrocarbons, notably *n*-hexadecane, are a poor criterion of purity. This phenomenon has been studied in the past; Broadhurst [14] has reviewed these studies and developed a general qualitative theory of solid phase behavior of normal paraffin hydrocarbons.

An unusual behavior was also encountered in the case of the melting point of the *n*-hexylbenzene. A number of melting points were determined of the same or similar samples, no two of which gave the same value. The following are values on one fraction: 1. one sample, −63.484° and −63.566 °C three days apart; 2. another sample, −63.502° and −63.538 °C the same day, one in the morning and one in the afternoon. It is possible that a solid phase transition takes place in the immediate vicinity of freezing (melting) point.

## Figures and Tables

**Table 1:** Distillation of cyclohexylbenzene Charge: 3700 ml of crude cyclohexylbenzene

Cut	Fraction No.	Head temperature °C at 60 mm Hg	Refractive index at 20 °C	Volume
				
				*ml*
A	1–7	146.4–156.5	1.5174–1.5226	314
B	7–14	156.5–156.6	1.5228–1.5244	356
C	15–22	156.6–156.6	1.5246–1.5253	304
D (a)	23–34	156.6–156.6	1.5251–1.5256	354
E	35–39	155.8–155.9	1.5262–1.5262	259
F	40–72	155.9–156.2	1.5262–1.5263	1663
G (b)	73	156.2–159.5	1.5285	42

a Distillation interrupted.

b The distillation was terminated because biphenyl clogged the takeoff and condenser.

**Table 2:** Physical properties of hydrocarbons prepared

Hydrocarbon	Hydrocarbon class	Freezing point	Boiling point at 760 mm Hg	dt/dp at 760 mm Hg	Refractive index		Density		Purity
					at 20° C	at 25° C	at 20° C	at 25° C	
									
		*°C*	*°C*	*°C/mm*	n_D_	n_D_	*g/ml*	*g/ml*	*mole percent*
Biphenyl	Bicyclic aromatic	+68.95	………	………	………	………	………	………	99.96
Cyclohexylbenzene	Naphthenic aromatic	+6.99	240.12	0.0525	1.52633	1.52393	0.94272	0.93874	99.83
Bicyclohexyl	Bicyclic naphthene	+3.63	239.04	.0555	1.47995	1.47768	.88619	.88249	99.96
*n*-Hexylbenzene	Alkylaromatic	a −63.5	225.77	.0563	1.48668	1.48445	.85802	.85428	(b)
*n*-Hexylcyclohexane	Naphthene	−47.51	224.78	.0572	1.44690	1.44459	.80817	.80452	(b)
1-Cyclopentylkeptane	Naphthene	−61.04	223.92	.0560	1.44231	1.43993	.80132	.79778	(b)
*n*-Dodecane	Paraffin	−9.71	216.26	.0557	1.42174	1.41955	.74901	.74535	99.52

(a) See section 3 of text.

(b) These hydrocarbons were analyzed by vapor-phase chromatography. In each case only one peak was noted.

## Footnotes

2 Figures in brackets indicate the literature references at the end of this paper.

3 Distillation Products Industries, Rochester, New York.

4 Matheson, Coleman & Bell Division, The Matheson Co., Inc., East Rutherford, N.J.
